# The Effect of 16 Weeks of Lower-Limb Strength Training in Jumping Performance of Ballet Dancers

**DOI:** 10.3389/fphys.2021.774327

**Published:** 2022-01-12

**Authors:** Lurdes Ávila-Carvalho, Filipe Conceição, Juan A. Escobar-Álvarez, Beatriz Gondra, Isaura Leite, Luís Rama

**Affiliations:** ^1^Faculty of Sports Science and Physical Education, University of Coimbra, Coimbra, Portugal; ^2^Faculty of Sport, Center of Research, Education, Innovation and Intervention in Sport (CIFI2D), University of Porto, Porto, Portugal; ^3^LABIOMEP, Porto Biomechanics Laboratory, University of Porto, Porto, Portugal; ^4^School of Health and Life Sciences, University of the West of Scotland, Ayr, United Kingdom; ^5^Research Center for Sport and Physical Activity (CIDAF), Faculty of Sports Science and Physical Education, University of Coimbra, Coimbra, Portugal

**Keywords:** jump, explosive, strength, power, dance

## Abstract

Jumping ability is considered a determinant of performance success. It is identified as one of the predictors and talent identification in many sports and dance. This study aimed to investigate the effect of 16 weeks of lower-limb strength training on the jumping performance of ballet dancers. A total of 24 participants from the same dance school were randomly selected in the control group [CG; *n* = 10; aged 13.00 (1.49) years; 43.09 (9.48) kg and 1.53 (0.11) m] and the intervention group [IG; *n* = 14; aged 12.43 (1.45) years; 38.21 (4.38) kg and 1.51 (0.07) m], evaluated before and after the applied strength training program mainly using the body weight of each participant. Jump performance was assessed using MyJump2, a scientifically validated mobile phone app. Intergroup and intragroup comparisons were assessed, and the magnitude of change was calculated using the effect size (ES). While CG significantly decreased the relative power over time (*p* < 0.001, ES = −0.29: small), results from the intragroup comparisons suggest that IG significantly increased the countermovement jump (CMJ) height (*p* < 0.001, ES = 1.21: large), the relative force (*p* < 0.001, ES = 0.86: moderate), maximal velocity (*p* < 0.001, ES = 1.15: moderate), and relative power (*p* < 0.001, ES = 1.37: large). We concluded that a 16-week strength training program of lower limbs is an effective way to improve CMJ height in young dancers. Supplementary strength training appears to be the determinant for the improvement of the jumping performance of ballet dancers.

## Introduction

Both sports and dance culminate in public performances. The intersections of dance and sports are reported since some athletes and coaches use strength training to improve their performance (Markula, 2018), particularly in aesthetic sports such as figure skating and gymnastics. In addition to the dramatic and artistic performance of dancers, dance demands high levels of motor performance, which include a high jumping ability (Koutedakis et al., 2005). Several authors recognize that dancers with a more developed jumping ability also improve the perception of aesthetic and artistic components of the choreography (Wyon et al., 2006; Angioi et al., 2009; Rafferty, 2010). Others underline the importance of jumping ability in talent identification in dance (Walker et al., 2010).

Studies involving dance students report that the increase in strength is crucial for the dancers to perform well, especially concerning the use of the lower limbs (essential in jumping) (Fração et al., 1999). In fact, given the abundance of ballistic actions in ballet (e.g., jumps and changes of direction), improvements in jump height may be beneficial for the dancers as a guide to specific training plans that can improve either maximal force or velocity capabilities (Alvarez et al., 2020). The countermovement jump (CMJ) is usually used to evaluate the power output of the lower limbs as ballistic movements that include both concentric and eccentric phases closer to the sports and ballet movements. In fact, the CMJ and drop jump performances are related to *grand jeté* leap performance in dancers with different skill levels, being considered useful tools for monitoring the power-generating capacity of the lower body of the dancer, thus giving insight into the overall jumping capacity of the dancer (Blanco et al., 2019). Consequently, both strength and speed development are fundamental to increasing jumping ability (Jimenez-Reyes et al., 2014).

Some studies observed the effect of training programs to improve jumping performance, either in dancers or in rhythmic gymnastics, which is the gymnastics discipline more equivalent to dance (Wang et al., 2010; Piazza et al., 2014; Komeroski et al., 2016; Mlsnová and Luptáková, 2017; Tsanaka et al., 2017; Dobrijević et al., 2018; Skopal et al., 2020; Stošić et al., 2020). Focusing on dance, previous studies have applied for a specific strength training program, for 9 weeks, based on and adjusted according to the force-velocity profile of each dancer (Escobar-Alvarez et al., 2019); evaluated whether a 9-week resistance training program could have a significant effect on the strength and power of the lower limbs in adolescent dancers (Dowse et al., 2020); applied, for 10 weeks, a modern and recreational dance exercise program and trunk and leg muscle strengthening exercises in university dance students (Stošić et al., 2020) and used their ballet classes, modified with a focus on lower-limb strength (reduction in bar duration (from 45′ to 20′) and the *petit* and *grand allegro* exercises at the beginning of center work, for 8 weeks, as an intervention to analyze jumping ability (Tsanaka et al., 2017). Regarding the results about the jump height, four studies (Tsanaka et al., 2017; Escobar-Alvarez et al., 2019; Dowse et al., 2020; Stošić et al., 2020) obtained positive results with significant differences, i.e., the applied training promoted improvements in the vertical jump height of the dancer. Concerning the instrument, two studies have used MyJump2 with ballet dancers, but only one conducted an intervention (Escobar-Alvarez et al., 2019; Alvarez et al., 2020). These findings of the authors suggest that the experimental group presented significant differences with large effect sizes (ESs) in CMJ height and other jumping performance variables, namely, the theoretical maximal force (Escobar-Alvarez et al., 2019). Additionally, measurements of the distance covered by the center of mass during push-off are highlighted (Samozino et al., 2008, 2012, 2014) to control the growth process of the participants over time.

Despite the known technical and physical demands of elite dance, traditionally, strength training has not been considered important to the ongoing development of adolescent dancers (Dowse et al., 2020). We found only one study that reported an intervention program aiming to improve the jumping performance of younger dancers using traditional external loads (Dowse et al., 2020). Our study aimed to promote strength training mainly using the body weight of an individual. We hope that this will raise the awareness of the dance teachers toward the strength training benefits to enhance the jumping performance of the dancers. Therefore, this study provides an example of a training program without any equipment that could be applied in dance classes of young ballet dancers.

This study aimed to investigate the effect of 16 weeks of lower-limb strength training in the jumping performance of classic ballet dancers. For this, we (1) evaluated the mechanical variables during the CMJ of dancers, (2) proposed a specific training program, for 16 weeks, to improve the jumping performance, and (3) compared the mechanical variables during the CMJ of dancers before and after a specific jump training program application. We hypothesized that the proposed training program will positively affect the CMJ height of the dancers.

## Materials and Methods

The following sample inclusion criteria were established: (1) enrollment at the educational institution participating in the study; (2) absence of injury that prevented them from training in the last 3 months, during the intervention and at the time of evaluation; (3) no involvement in any complementary physical training or sports activity during the 16 weeks of intervention; and (4) attendance at a minimum of 80% of training sessions.

Table 1 shows the sample characterization of the control group (CG) and the intervention group (IG).

**TABLE 1:** Sample characterization [Mean (*SD*)].

Variables	CG (*n* = 10) mean (*SD*)	IG (*n* = 14) mean (*SD*)	*p*
Age (years)	13.00 (1.49)	12.43 (1.45)	0.357
Weight (kg)	43.09 (9.48)	38.21 (4.38)	0.156
Height (m)	1.53 (0.11)	1.51 (0.07)	0.487
BMI (kg/m^2^)	18.05 (2.46)	16.77 (1.02)	0.148
HPO (cm)	0.36 (0.07)	0.39 (0.07)	0.348
Years of practice	5.00 (1.83)	6.50 (3.52)	0.189
Hours/training/week	28.50 (3.37)	27.93 (5.14)	0.762

*p ≤ 0.05, independent measures t-test; pretest between groups.*

*CG, control group; IG, intervention group; BMI, body mass index; HPO, height of push-off; SD, standard deviation.*

Our sample was composed of 24 ballet dancers from the same school dance and was divided into the CG (*n* = 10) and the IG (*n* = 14). Although the participants were selected randomly, the groups did not have the same number of subjects because four dancers from the CG were involved in a complementary sports activity, which led to their exclusion from the CG sample. The sample size was restricted to the number of higher-level dancers from the dance school that accepted and approved to carry out this study.

It is essential to highlight that the CG was composed of 9 female and 1 male dancers, with an average age of 13.00 (1.49) years, 5 years of regular dance practice, and 28.50 h of training per week (Table 1). The IG was composed of 10 female and 4 male dancers, with an average age of 12.43 (1.45) years, 6.50 years of practice, and 27.93 h of weekly training. No significant differences were found between the CG and the IG for any of the variables in Table 1.

All ethical procedures were carried out following the Declaration of Helsinki, and the study was approved by the local Ethical Committee (CE/FCDEF-UC/00742021). All participants and their guardians (dancers aged under 18 years) were informed of the benefits and risks of the research. Before the beginning of the study, they signed an informed consent document for testing, implementation of the training protocol, and publication of collected data.

The variables assessed in this investigation were the weight (kg), height (m), body mass index (BMI) (kg/m^2^), CMJ (cm), relative force (N/kg), maximal velocity (m/s), relative power (W/kg), and the height of push-off (HPO) (cm) of each dancer. The relative values to body mass were calculated by dividing the output of each subject by their mass as previous findings, whereas relative force was calculated by dividing the force output of each subject by their mass (Dowse et al., 2020).

These mechanical variables were used in our study to better understand the CMJ high performance as stated by Cormie et al. (2011) that force and velocity are considered as the fundamental features of mechanical power output in sport movements.

Before the beginning of the study, the body weight (kg) and the stature (m) were collected using a Tanita SC-330 (TANITA Corp, Tokyo, Japan) and an aluminum stadiometer (Seca 713 model; Seca, Postfach, Germany), respectively. HPO assessment (Samozino et al., 2008, 2012, 2014) implicates two measurements of lower-limb length (in centimeters) by an experienced researcher using a tape measure (SECA, 201). First, with the participant lying down and the ankle fully extended, the distance from the iliac crest to toes and, second, squatting at 90° (knee flexion) from the iliac crest to the ground were measured. These measures were collected before each of the 3 evaluation moments to control the growth process of dancers along the 16 weeks of training. The time-lapse between the last training session and the evaluation moments was always proximally 72 h. Both groups were evaluated in pretest and after the 16 weeks of training. Since the IG experienced an unusual training strategy, an intermediated control moment of jump performance progression was programmed, which occurs at the 8th week only in IG.

Before all control time points, dancers performed their habitual warm-up routine consisting of 15 min of jogging, dynamic stretching (plantar flexors, hip extensors, hamstrings, hip flexors, and quadriceps femoris), and preparatory CMJs, also following previous orientations (Escobar-Alvarez et al., 2019). Before each jump, participants were instructed to remain in a standing position with their hands on their hips. From this position, participants performed a CMJ as described earlier (Jimenez-Reyes et al., 2014). A maximum effort CMJ was used to assess lower-body explosive power and the effect of the stretch-shorten cycle of each subject (Dowse et al., 2020). The instrument used was MyJump2, an app of iPhone 5 specially developed to monitor the vertical jump ability of the athlete in a valid, reliable, and economical way in adults (Balsalobre-Fernández et al., 2015; Jiménez-Reyes et al., 2017), and Samozino’s method was used to monitor children (Morin and Samozino, 2016; Bogataj et al., 2020). The analysis of jumping performance using MyJump2 evoked recently in scientific research (Samozino et al., 2012, 2014; Balsalobre-Fernández et al., 2015; Jimenez-Reyes et al., 2016; Morin and Samozino, 2016). This method is based on the fundamental laws of mechanics, which proposes an accurate and reproducible field method to evaluate the power output of lower limbs and allows a precision similar to that obtained with specific laboratory ergometers (force platform method) (Samozino et al., 2008). This instrument can be used to monitor the performance of the athletes and dancers without expensive laboratory equipment or moving the athletes and dancers from their usual practice zone. It allows assessing the external force developed and the maximum speed capacity related to body mass (Jimenez-Reyes et al., 2014; Samozino et al., 2014; Jiménez-Reyes et al., 2017), thus personalizing the results to the characteristics of individual athletes or dancers.

Both groups (IG and CG) maintained the standardized training regimen (as presented in Supplementary Table 1). In addition, the IG followed a program of the lower-limb strength training session two times a week during the 16 weeks of intervention. Dancers performed a training program (20 min) mainly based on exercises using their body weight, following previous recommendations for youth training (Faigenbaum et al., 2009). The training program had four phases: phase 1 (weeks 1–4) was composed of full squat, single-leg squat, and step-up exercises; phase 2 (weeks 5–10) was composed of introducing box jumps, single-leg jumps, burpees, and lunges step-ups; phase 3 (weeks 11–13) was composed of Russian squats in pairs, bouncing, CMJ, and lateral step-ups; and phase 4 (week 14–16) was composed of isometric squats, single-leg squat jumps, leg press in pairs, and CMJ. Details about repetitions, sets, recovery, and duration of each phase are presented in Supplementary Table 2.

All data are presented as means (SDs) using IBM SPSS Statistics for Windows, Version 27.0. Armonk, NY, United States. The normal distribution of the study variables was assessed using the Shapiro–Wilk test. Intergroup and intragroup comparisons were evaluated by an independent measure and a repeated measures *t*-test, respectively. We also conducted a repeated-measures ANOVA with Bonferroni adjustment (3 evaluation moments) to include a data collection performed at the 8th week only at the IG as a control measure of jump performance evolution. The level of significance was set at *p* ≤ 0.05.

Intragroup magnitudes of change were calculated with the following S (Hopkins, 2004). The criterion for interpreting these magnitudes was as follows: < 0.2, trivial change; 0.2–0.6, small; 0.6–1.2, moderate; 1.2–2, large; > 2.0, very large (Hopkins et al., 2009). The probability that these differences exist was assessed *via* magnitude-based qualitative inferences (Batterham and Hopkins, 2006). Probabilities that differences were higher than, lower than, or similar to the smallest worthwhile difference were defined by the following scale: < 0.5%, almost certainly not; < 5%, very unlikely; < 25%, unlikely, probably not; 25%–75%, possibly, possibly not; > 75%, likely, probably; > 95%, very likely; > 99.5%, almost certainly. Finally, for the intergroup comparison, we used the ES from Cohen’s *D*, using the following scale for interpretation: 0.2–0.5, small; 0.5–0.8, moderate; > 0.8, large (Cohen, 1988).

## Results

Although our sample is composed of female and male dancers, similar to a previous study (Dallas et al., 2014), no significant differences (*p* ≥ 0.05) were found between the sexes in the jumping performance. Accordingly, we considered them as one group for the study analysis. Table 2 shows the intergroup comparison (CG and IG) of the jumping performance variables during the pre-post intervention.

**TABLE 2:** Intergroup comparison of jumping performance variables pre-post intervention.

Variables	CG pretest (*N* = 10) mean (*SD*)	IG pretest (*N* = 14) mean (*SD*)	*p*	ES	CG posttest (*N* = 10) mean (*SD*)	IG posttest (*N* = 14) mean (*SD*)	*p*	ES
Weight (kg)	43.09 (9.48)	38.21 (4.38)	0.156	0.704	43.46 (9.69)	40.21 (4.56)	0.345	0.456
Height (m)	1.53 (0.11)	1.51 (0.07)	0.487	0.293	1.54 (0.11)	1.52 (0.07)	0.657	0.187
BMI (kg/m^2^)	18.05 (2.46)	16.77 (1.02)	0.148	0.728	18.07 (2.42)	17.32 (1.08)	0.377	0.428
CMJ (cm)	29.33 (5.73)	25.10 (3.70)	0.039*	0.911	28.60 (6.24)	29.85 (3.43)	0.534	−0.261
Relative force (N/kg)	17.74 (0.96)	16.38 (1.07)	0.004*	1.326	17.19 (1.76)	17.36 (1.09)	0.773	−0.121
Maximal velocity (m/s)	1.20 (0.11)	1.11 (0.08)	0.035*	0.931	1.18 (0.12)	1.21 (0.07)	0.460	−0.311
Relative power (W/kg)	21.25 (2.80)	18.14 (1.96)	0.004*	1.328	20.89 (2.96)	20.99 (2.01)	0.923	−0.041

CMJ, countermovement jump; ES, effect size (Cohen, 1988). *p ≤ 0.05.

In pretest, the CG presented significantly higher CMJ (*p* = 0.039, ES = 0.911: large), relative force (*p* = 0.004, ES = 1.326: large), maximal velocity (*p* = 0.035, ES = 0.931: large), and relative power values (*p* = 0.004, ES = 1.328: large) in comparison to the IG. In posttest, no significant differences were found between groups in any of the study variables.

Table 3 shows the intragroup comparisons for anthropometric and jumping performance variables.

**TABLE 3:** Intragroup comparison of anthropometric and jumping performance variables pre-post intervention.

		Variables	Weight (kg)	Height (m)	BMI (kg/m^2^)	CMJ (cm)	Relative force (N/kg)	Maximal velocity (m/s)	Relative power (W/kg)
CG (*n* = 10)		Pretest mean (*SD*)	43.09 (9.48)	1.53 (0.11)	18.05 (2.46)	29.33 (5.73)	17.74 (0.96)	1.20 (0.11)	21.25 (2.80)
		Posttest mean (*SD*)	43.46 (9.69)	1.54 (0.11)	18.07 (2.42)	28.60 (6.24)	17.19 (1.76)	1.18 (0.12)	20.89 (2.96)
		p	0.069	0.096	0.574	0.273	0.256	0.263	<0.001*
		ES	0.04 (0.03)	0.04 (0.04)	0.01 (0.03)	−0.12 (0.18)	−0.48 (0.72)	−0.14 (0.21)	−0.29 (0.42)
		95% CL	0, 0.07	0, 0.08	−0.02, 0.04	−0.3, 0.07	−1.2, 0.24	−0.35, 0.07	−0.71, 0.13
		Inference	Trivial	Trivial	Trivial	Trivial	Small	Trivial	Small
	Individual response	Probability	Almost certainly	Almost certainly	Almost certainly	Likely	Possibly	Possibly	Possibly
		Positive-trivial-negative	0-100-0	0-100-0	0-100-0	1-78-21	6-19-75	1-69-30	3-32-65
IG (*n* = 14)		Pretest mean (*SD*)	38.21 (4.38)	1.51 (0.07)	16.77 (1.02)	25.10 (3.70)	16.38 (1.07)	1.11 (0.08)	18.14 (1.96)
		Posttest mean (*SD*)	40.21 (4.56)	1.52 (0.07)	17.32 (1.08)	29.85 (3.43)	17.36 (1.09)	1.21 (0.07)	20.99 (2.01)
		p	<0.001*	<0.001*	0.003*	<0.001*	<0.001*	<0.001*	<0.001*
		ES	0.43 (0.13)	0.19 (0.07)	0.51 (0.24)	1.21 (0.26)	0.86 (0.22)	1.15 (0.25)	1.37 (0.3)
		95% CL	0.3, 0.56	0.12, 0.26	0.27, 0.75	0.95, 1.47	0.64, 1.08	0.9, 1.4	1.07, 1.67
		Inference	Small	Trivial	Small	Large	Moderate	Moderate	Large
	Individual response	Probability	Almost certainly	Likely	Almost certainly	Almost certainly	Almost certainly	Almost certainly	Almost certainly
		Positive-trivial-negative	100-0-0	39-61-0	98-2-0	100-0-0	100-0-0	100-0-0	100-0-0

95% CL, 95% confidence limits. *p ≤ 0.05 (significant differences); ES – effect size (Hopkins, 2004).

The CG significantly decreased relative power values over time (*p* < 0.001). In contrast, the IG significantly increased all variables, namely, the anthropometric measurements, weight (*p* < 0.001, ES = 0.43: small), height (*p* < 0.001, ES = 0.19: trivial) and BMI (*p* ≤ 0.003, ES = 0.51: small), CMJ (*p* < 0.001, ES = 1.21: large), relative force (*p* < 0.001, ES = 0.86: moderate), maximal velocity (*p* < 0.001, ES = 1.15: moderate), and relative power (*p* < 0.001, ES = 1.37: large).

Table 4 presents the intragroup comparison in the IG dancers during the 3 evaluation moments (initial, after 8 weeks, and after 16 weeks of training protocol).

**TABLE 4:** Intervention group, intragroup comparison (3 evaluation moments).

Variables	N	Pretest mean (*SD*)	Posttest mean (*SD*)	Posttest2 mean (*SD*)	*p* (Wilks Lambda)	*Post hoc* (Bonferroni)
Weight (kg)	14	38.21 (4.38)	38.78 (4.34)	40.21 (4.56)	<0.001*	All moments
Height (m)	14	1.51 (0.07)	1.51 (0.07)	1.52 (0.07)	0.002*	Moments 1–3, 2–3
BMI (kg/m^2^)	14	16.77 (1.02)	16.95 (1.05)	17.32 (1.08)	0.010*	All moments
CMJ (cm)	14	25.10 (3.70)	28.03 (3.82)	29.85 (3.43)	<0.001*	All moments
Relative force (N/kg)	14	16.38 (1.07)	17.01 (0.98)	17.36 (1.09)	<0.001*	Moments 1–2, 1–3
Maximal velocity (m/s)	14	1.11 (0.08)	1.17 (0.08)	1.21 (0.07)	<0.001*	All moments
Relative power (W/kg)	14	18.14 (1.96)	19.92 (2.06)	20.99 (2.01)	<0.001*	All moments

*p ≤ 0.05.

Finally, Table 4 presents an intermediate evaluation moment, including the IG dancers as a control measure of jump performance evolution. It indicates that the anthropometric variables significantly increased over time, but the height of the dancer only differs significantly between moments 1–3 and 2–3 (*p* = 0.002). Regarding the jumping performance, CMJ (*p* < 0.001), maximal velocity (*p* < 0.001), and relative power (*p* < 0.001) increased significantly and progressively across all evaluation moments. Finally, relative force increased significantly between moments 1–2 and 1–3 (*p* < 0.001).

## Discussion

This study aimed to investigate the effect of 16 weeks of lower-limb strength training on the jumping performance of ballet dancers. We have compared the jumping performance variables during the CMJ of dancers before and after a training program, for 16 weeks. Our findings confirm the hypothesis formulated initially since the training program positively affected the CMJ height of the dancers. While the CG significantly decreased relative power values over time (*p* < 0.001), intragroup comparisons indicate that the IG significantly increased the CMJ height (*p* < 0.001, ES = 1.21: large), relative force (*p* < 0.001, ES = 0.86: moderate), maximal velocity (*p* < 0.001, ES = 1.15: moderate), and relative power (*p* < 0.001, ES = 1.37: large) after the 16-week training program. The ES interpretation and the individual response results suggest that these improvements in CMJ, relative force, velocity, and relative power represent almost certainly (100%) a positive effect from the training program (Table 3), which supports its efficiency.

In fact, our findings suggest that in pretest, the CG presented significantly higher CMJ (*p* = 0.039, ES = 0.911: large), relative force (*p* = 0.004, ES = 1.326: large), maximal velocity (*p* = 0.035, ES = 0.931: large), and relative power values (*p* = 0.004, ES = 1.328: large) in comparison to the IG. Regarding the CMJ, this advantage of the CG over the IG in pretest [29.33 (5.73) cm vs. 25.10 (3.70) cm, respectively] with a large ES is in opposition with the previous findings where the IG presented an initial advantage in this variable (Escobar-Alvarez et al., 2019). Besides this initial advantage (of 4.23 cm in comparison to the IG), the intragroup comparisons indicate that the training program allowed the IG to increase this variable by 4.75 cm [achieving 29.85 (3.43) cm]. In contrast, the CG jump height declined to 28.60 (6.24) cm after the 16 weeks, despite previous findings indicating minimal changes in CG jump height over time [e.g., 27.3 (2) cm vs. 27.5 (2) cm] (Escobar-Alvarez et al., 2019).

Previous studies that observed the effect of training programs in the jump height of dancers have also obtained improvements in this variable with values pre-post diverging between 16.9 (2.9) cm and 18.9 (2.7) cm (*p* < 0.001, *d* = 0.36: small ES) pre-post 30 weeks of a plyometric training program and in CMJ with arm swing dancers jumped 21.5 (3) cm vs. 25 (2.8) cm (*p* < 0.001, d = 1.21: large), an ES equal to our findings in CMJ (Mlsnová and Luptáková, 2017); 22.50 (4.21) cm and 25.47 (4.95) cm pre-post a 10-week modern and recreational dance exercise program and trunk and leg muscle strengthening exercises (Stošić et al., 2020); 26.93 (2.78) cm and 27.35 (3.06) cm pre-post an 8-week protocol (Tsanaka et al., 2017); and 29.3 (3.2) cm and 33.5 (3.7) cm with a significant improvement (Escobar-Alvarez et al., 2019) aligned with our findings. Still, in studies without interventions, CMJ height values ranged between 23.34 (1.72) cm in dancers aged 15 (1.07) years (Rojano-Ortega, 2020) and 28.29 (3.42) cm in dancers aged 18.94 (1.32) years (Alvarez et al., 2020). In comparison, in this study, the average age of IG dancers was 12.43 (1.45) years and the pre-post CMJ height ranged between 25.10 (3.70) cm and 29.85 (3.43) cm, underlining the proficiency of our training program in the jump height of IG dancers.

Regarding the significant improvements in the IG relative force (16.38 (1.07) N/kg vs. 17.36 (1.09) N/kg, *p* < 0.001, ES = 0.86: moderate) and relative power (18.14 (1.96) W/kg vs. 20.99 (2.01) W/kg, *p* < 0.001, ES = 1.37: large), and since our sample was composed of younger dancers, it was important to calculate these variables relative to body mass of each dancer from simple computation measures based on body mass, jump height (from flight time), and push-off distance (Jiménez-Reyes et al., 2017). A previous study obtained a significant improvement in lower-body peak force after a resistance training program in adolescent dancers (Dowse et al., 2020). We observed that our relative power values are lower than those observed in other studies with older dancers, such as 27.14 (1.80) W/kg (Rojano-Ortega, 2020) and values pre-post an intervention in rhythmic gymnastics ranging from 21.66 (4.09) W/kg to 23.98 (4.48) W/kg with a 9.67% improvement according to the authors (Grande Rodríguez et al., 2010). Although we also obtained significant improvements in the IG maximal velocity [1.11 (0.08) m/s vs. 1.21 (0.07) m/s, *p* < 0.001, ES = 1.15: moderate], previous studies have also obtained higher values, such as 2.30 (0.12) m/s vs. 2.31 (0.13) m/s in dancers (Tsanaka et al., 2017) and 2.33 (0.18) m/s vs. 2.45 (0.23) m/s in rhythmic gymnastics (Grande Rodríguez et al., 2010). Previous findings suggest that dance training mainly develops velocity capabilities, and supplemental force training may be beneficial regarding the high number of dramatic elevations that dance performance requires (Alvarez et al., 2020).

As stated previously, we controlled the growth of the dancers over the 16 weeks of training using HPO measurements. Still, younger dancers are inevitably in maturation and growing processes, similar to previous studies (Dowse et al., 2020), which may be a possible justification for these dissimilarities. Additionally, female and male dancers did not differ significantly in the jumping performance variables (*p* ≥ 0.05). The apparent sample homogeneity could be explained by the maturation process, whereas girls could be at a higher maturity stage and balanced the performance of the boys. Accordingly, we considered them as one group for the study analysis.

Since we could not measure the jumping performance every 3 weeks during the program, and 1 week after the end of the program as recommended (Jiménez-Reyes et al., 2019), the additional evaluation moment at week 8, precisely in the middle of the protocol, represented a control measure of the IG jump performance evolution. After 8 weeks of avoiding external load and promoting working with the bodyweight of an individual as much as possible following the recommendations for youth training Faigenbaum et al. (2009), the training program included exercises where dancers had to overcome the strength of a partner to perform specific exercises (e.g., leg press with the partner sitting on their feet, see Supplementary Material). This adjustment to the training program reveals that an external resistance may be an essential aid in improving the jumping performance of dancers (Escobar-Alvarez et al., 2019). Additionally, it discloses the importance of controlling the training and adapting it to the individual needs of the athlete (Alvarez et al., 2020), that is, of including intermediate evaluation moments within the training program applications in scientific research; considering both training content and training duration together may enable more individualized, specific, and effective training monitoring and periodization (Jiménez-Reyes et al., 2019).

The training program of this study included exercises that develop the strength component, exercises that stimulated the explosive strength, and ballistic exercises that correspond to the stretch-shortening cycle action (Escobar-Alvarez et al., 2019). Although not significant, the CG CMJ height, relative force, and maximal velocity values decreased and the relative power decreased significantly (*p* < 0.001, ES = − 0.29: small) during the 16 weeks with standard ballet classes. These findings clearly correspond to previous research suggesting that dance training alone may not provide sufficient overload to evoke a physiological change in adolescent dancers and identified that the inclusion of a strength training program facilitated improvements in maximum lower-body strength (Koutedakis et al., 2007) and vertical CMJ height (Brown et al., 2007). While the literature provides contradictory results regarding the determination of the optimal plyometric volume to enhance jumping performance, two previous studies suggest that a low volume in plyometric jumps can lead to a higher increase in CMJ (Chen et al., 2013; Baena-Raya et al., 2019). In contrast, a previous meta-analysis stated that training protocols based on jumps (plyometric) or resisted training weightlifting exercises provide similar results in jumping performance (Berton et al., 2018).

In fact, the significant and progressive increment over all of the evaluation moments of the CMJ (*p* < 0.001) is aligned with previous outcomes that also conducted additional evaluation moments (Escobar-Alvarez et al., 2019), suggesting that these protocols are an effective way to improve CMJ height in female ballet dancers. This is a significant step forward for the dance conditioning literature and provides a platform for research and practice in dance-specific additional training (Véliz et al., 2016).

Other studies also showed significant improvements in the jumping performance of dancers (Wang et al., 2010; Komeroski et al., 2016; Tsanaka et al., 2017; Escobar-Alvarez et al., 2019; Stošić et al., 2020) and rhythmic gymnasts (Piazza et al., 2014; Dobrijević et al., 2018; Dallas et al., 2020) as a result of training interventions, and the improvements in jumping performance are consistent with previous findings in ballet dancers (Escobar-Alvarez et al., 2019; Alvarez et al., 2020) and in other sports disciplines (Jimenez-Reyes et al., 2016, Jiménez-Reyes et al., 2019). We suggest an exercise prescription based on the individual needs and the physical demands of ballet, jazz, and contemporary dancers as referred to in previous studies (Alvarez et al., 2020; Dowse et al., 2020).

Our results are aligned with previous findings, suggesting that incorporating resistance training may enhance strength and power adaptations in adolescent dancers, which can be achieved with minimal equipment and can be performed in the training space of the dancers, as our previous design of the training programs indicates (Dowse et al., 2020; Skopal et al., 2020). Our findings support earlier recommendations regarding integrating resistance training methods (Véliz et al., 2016; Tsanaka et al., 2017) or strength and conditioning coaches (Tsanaka et al., 2017) to deliver systematic resistance training to adolescent dancers. Other authors also refer that this may facilitate skill acquisition during growth and reduce the potential for injury (Brown et al., 2007) and that the inclusion of strength training may be able to manage growth and maturational-related changes that commonly lead to decrements in strength, balance, and the ability to master dance-specific technical skills (Daniels et al., 2001). Furthermore, by demonstrating the potential for adaptation within an adolescent cohort, it is hoped that this will increase the awareness of the strength training benefits and encourage dancers and support staff to consider a more integrated approach to training (Dowse et al., 2020).

We recognize that our study presents some limitations, such as a reduced sample size in IG and CG, including only ballet dancers. It is not known whether the results are generalizable to other dance styles. We also acknowledge that our sample was formed by younger dancers who were in the maturation process. Although we did not control the maturity of the participants, the growth process was perceived by the HPO measure, since it would have a direct influence on the validity and reliability of the instrument used. This marker did not show a significant modification along the intervention period, which led the authors to assume maturity stability during the study. We also included female and male participants, which can influence the results. Lastly, the evaluation of the transference of improvement in jump height in a specific dance skill was not conducted.

## Conclusion and Practical Implications

A 16-week training program of lower-limb strength training using mainly own body mass effectively improve CMJ height in young dancers and can present practical implications for dance training. Supplementary strength training seems to be effective for improving jumping performance in ballet dancers.

We suggest that the incorporation of 20 min of strength and plyometric additional training could improve the jump height of the ballet dancers.

The design of the training program suggests that this is possible with no equipment and may be easily incorporated in the dance training schedule and the typical dancer’s training space.

## Future Research

We suggest more investigation in this area, seeking a better understanding of the dance physical needs, making more information available for dance professors to better complement their training programs. Future studies should aim for a more individualized, specific, and effective training monitoring and periodization (e.g., variables measured every 3 weeks during the program and every week after the end of the individualized program) (Jiménez-Reyes et al., 2019). It would be important to assess if the study results could be transferred to perform ballet-specific skills.

## Data Availability Statement

The raw data supporting the conclusions of this article will be made available by the authors, without undue reservation.

## Ethics Statement

The studies involving human participants were reviewed and approved by the Ethical Committee, Faculty of Sports Science and Physical Education, University of Coimbra (CE/FCDEF-UC/00742021). Written informed consent to participate in this study was provided by the participants’ legal guardian/next of kin.

## Author Contributions

LÁ-C, FC, JE-Á, and LR participated in study design, data collection, and the writing of the first draft manuscript. LÁ-C, FC, JE-Á, BG, IL, and LR participated in the article collection and analysis. LÁ-C, FC, JE-Á, IL, and LR participated in the writing of the methodology and results, and the final revisions of the manuscript. All authors have read and approved the final version of the manuscript and agreed with the order of presentation of the authors.

## Conflict of Interest

The authors declare that the research was conducted in the absence of any commercial or financial relationships that could be construed as a potential conflict of interest.

## Publisher’s Note

All claims expressed in this article are solely those of the authors and do not necessarily represent those of their affiliated organizations, or those of the publisher, the editors and the reviewers. Any product that may be evaluated in this article, or claim that may be made by its manufacturer, is not guaranteed or endorsed by the publisher.
