# Supplementary material for: The Effect of 16 Weeks of Lower-Limb Strength Training in Jumping Performance of Ballet Dancers
Source: Front Physiol. 2022 Jan 12;12:774327. doi: 10.3389/fphys.2021.774327 (PMC8790119; doi:10.3389/fphys.2021.774327)
Supplement: Supplementary file 1 [file Table_1.docx]

**Supplementary table 1: Model of regular dance training**

| **Classes** | **Content** | **Daily training** |
| --- | --- | --- |
| Warm-up | Jogging, dynamic stretching, preparatory jumps. | 15 minutes |
| Physical Preparation | Dynamic and passive stretching; Coordination work;  General Plyometric exercises; Arms, legs, abdominal and back general strength work. | 30 minutes |
| Classical Class | Barre exercises. | 30 minutes |
|  | Center exercises:  Adagio,  Pirouettes- turns,  Allegro,  Point Work. | 1 hour |
| Repertoire/rehearsal | Solos/*Pas de Deux*/Groups | 1 hour |
| Contemporary Class | Technique, | 1 hour |
|  | Choreography. | 30 minutes |
