# Supplementary material for: The Effect of 16 Weeks of Lower-Limb Strength Training in Jumping Performance of Ballet Dancers
Source: Front Physiol. 2022 Jan 12;12:774327. doi: 10.3389/fphys.2021.774327 (PMC8790119; doi:10.3389/fphys.2021.774327)
Supplement: Supplementary file 2 [file Table_2.docx]

| Exercises | Repetitions | Series | Rest period (between exercises; between series) |
| --- | --- | --- | --- |
| Weeks 1-4 | | | |
| 1. Full squat with maximum execution in concentric phase | 10 | 3 | 1:30s; 30s |
| 2. Single leg squat (sitting on a bench) | 10 each leg | 1 | 1:30s; 30s |
| 3. Step-up | 10 each leg | 2 | No rest period between each limb |
| Weeks 5-10 | | | |
| 4. Box jump (adaptation – Squat Jump) | 8 | 3 | 1:30s; 30s |
| 5. Single leg jumps (6 jumps with each leg)^[[1]](#footnote-1)^ | Right and left | 3 | No rest period between each limb |
| 6. Burpees (hold squat position for 1-2s) | 6 | 3 | 1:30s/30s |
| 7. Lunges Step-ups^[[2]](#footnote-2)^ | 8 | 3 | 1:30s/30s |
| Weeks 11-13 | | | |
| 8. Russian squat with support of a partner | 5 | 1 | 1min30s between exercises |
| 9. Bouncing | Right and left | 2 | No rest |
| 10. CMJ | 8 | 2 | 1:30s/30s |
| 11. Lateral step up | 3 | 3 | 1:30s/30s |
| Weeks 14-16 | | | |
| 12. Isometry squat for 5s (opposition force made by a partner) followed by 4 vertical jumps | Week 14 – 2x; Week 15 – 3x; Week 16 – 2x | 1 | 1:30s/30s |
| 13. Single leg squat jump (rear leg hold by a partner) | Right and left | 6 | No rest |
| 14. Leg press with partner as additional load | 8 | 2 | 1:30s/30s |
| 15. CMJ | 8 | 2 | 1:30s/30s |

**Supplementary table 2: 16-week lower limb strength training program**

1. Jump a series of cones (6) in a straight line separated by a distance which allows to work on plyometrics continuously. Once they have mastered the technique, they can place their hands on the waist or hold a "bar"/stick (without weight). [↑](#footnote-ref-1)
2. Explosive movements combined with single leg jump after the lunge and landing with both legs according to the dancer’s progression. [↑](#footnote-ref-2)
